# Bark Beetles Utilize Ophiostomatoid Fungi to Circumvent Host Tree Defenses

**DOI:** 10.3390/metabo13020239

**Published:** 2023-02-06

**Authors:** Rashaduz Zaman, Courtney May, Aziz Ullah, Nadir Erbilgin

**Affiliations:** Department of Renewable Resources, University of Alberta, Edmonton, AB T6G 2E3, Canada

**Keywords:** *Dendroctonus ponderosae*, diterpenes, *Pinus contorta*, *Grosmannia clavigera*, *Leptographium longiclavatum*, monoterpenes, *Ophiostoma montium*, secondary metabolites, terpene detoxification

## Abstract

Bark beetles maintain symbiotic associations with a diversity of microbial organisms, including ophiostomatoid fungi. Studies have frequently reported the role of ophiostomatoid fungi in bark beetle biology, but how fungal symbionts interact with host chemical defenses over time is needed. We first investigated how inoculations by three fungal symbionts of mountain pine beetle affect the terpene chemistry of live lodgepole pine trees. We then conducted a complimentary laboratory experiment specifically measuring the host metabolite degradation by fungi and collected the fungal organic volatiles following inoculations with the same fungal species on lodgepole pine logs. In both experiments, we analyzed the infected tissues for their terpene chemistry. Additionally, we conducted an olfactometer assay to determine whether adult beetles respond to the volatile organic chemicals emitted from each of the three fungal species. We found that all fungi upregulated terpenes as early as two weeks after inoculations. Similarly, oxygenated monoterpene concentrations also increased by several folds (only in logs). A large majority of beetles tested showed a strong attraction to two fungal species, whereas the other fungus repelled the beetles. Together this study shows that fungal symbionts can alter host defense chemistry, assist beetles in overcoming metabolite toxicity, and provide possible chemical cues for bark beetle attraction.

## 1. Introduction

Bark beetles (Coleoptera: Curculionidae, Scolytinae) are subcortical insects that primarily feed on host tree phloem. These species play critical roles in maintaining the ecosystem function, including nutrient cycling, by killing defensively compromised trees (stressed, diseased, etc.). Host tree colonization starts with the release of aggregation pheromones by the pioneering beetles that attract conspecifics after locating a potentially suitable host tree. The aggregation pheromone is produced by bark beetles either *de novo* or using the host chemicals as precursors. During host colonization, beetles also introduce their symbiotic ophiostomatoid fungi into the host trees. All bark beetles are associated with several species of fungi from the genera *Ophiostoma*, *Ceratocystiopsis*, *Grosmannia*, or *Ceratocystis* [1]. These fungal symbionts are critical components of successful host-tree colonization by bark beetles [2,3,4]. After mating, female beetles excavate oviposition galleries and lay eggs. The newly hatched larvae make their galleries where they feed on phloem tissues infected with the fungal symbionts [5,6,7]. Due to their widespread associations [8,9,10,11,12], there is growing literature on bark beetle-fungal interactions; however, fungal-host tree interactions have received relatively less attention. In particular, how fungal infection alters the production of host secondary metabolites over time and their role in assisting beetles in overcoming metabolite toxicity require additional studies. Furthermore, ophiostomatoid fungi produce a diversity of fungal volatile organic compounds or FVOC [13,14,15,16]. In a relatively few species of bark beetles, the role of FVOCs in bark beetle attraction was reported [6,16,17,18].

Several species of bark beetles can also attack healthy trees once their populations reach a certain threshold density [19,20,21]. Such attacks usually lead to landscape-level tree mortality. However, coniferous trees have developed sophisticated defenses against bark beetles-fungi complexes that comprise physical, chemical, and histological mechanisms that can be expressed both constitutively and induced. The main constitutive response is located in the secondary phloem, which contains cells that act as mechanical barriers against attacking beetles. In particular, the resin cells produce oleoresin that contains terpenes that provide chemical protection against the bark beetle-fungal complexes [22]. At the induced phase, attacks induce resinosis and additional traumatic resin duct formation, auto-necrosis, and biosynthesis of structurally diverse terpenoids through the methylerythritol phosphate pathway [23,24,25]. Oleoresins are toxic to both beetles and fungi and also physically entrap invading bark beetles [26,27,28,29]. However, some monoterpenes are utilized by bark beetles as precursors for pheromone production during host colonization [27,30,31]. For instance, the Norway spruce beetle, *Ips typographus,* can oxidize host monoterpene α-pinene to *cis*-verbenol, which is then used as an aggregation pheromone by the same beetles in combination with fungal-produced volatile, 2-methyl-3-buten-2-ol [32,33].

The mountain pine beetle (MPB, *Dendroctonus ponderosae* Hopkins) is an eruptive native bark beetle species in western North America and has killed millions of pines, mainly lodgepole pine (*Pinus contorta* Douglas), during the last outbreak [21]. Three main symbiotic fungi associated with MPB include *Grosmannia clavigera* (Robinson-Jeffery and Davidson) Zipfel, de Beer, and Wing, *Ophiostoma montium* (Rumford) von Arx, and *Leptographium longiclavatum* Lee, Kim, and Breuril [34,35,36]. Toxic terpenes such as monoterpenes and diterpene resin acids are produced in response to MPB attacks [29,37].

The interaction between fungi growing in beetle-colonized hosts and the host defense chemistry may result in the production of metabolites that can be attractants to bark beetles [16,38]. Determining whether fungi can emit bark beetle-attractive compounds would promote our understanding of the sources of semiochemical (behavior modifying specific compounds) landscape that bark beetles encounter during host-tree colonization. Although several studies have shown the ability of symbiotic fungi to modify host defense chemistry, the mechanism of how bark beetles surpass host defenses in the MPB-symbiotic fungal complex is still not clear. Such understanding can help us to determine the symbiotic fungi’s role in aiding beetles to overcome the host defenses. We hypothesize that symbiotic fungi improve beetles’ successful host colonization (1) by modifying terpene defenses of trees and (2) by attracting beetles towards fungal volatiles that may signal favorable breeding-host substrates.

Several studies have investigated the role of FVOCs in fungus-tree, fungus-beetle, and fungus-fungus interactions [13,16,17,39,40,41,42,43]. These studies have reported that (1) host defense chemistry, mainly monoterpenes, can affect the production of FVOCs; (2) different fungal species have similar FVOC profiles, but the abundance of specific compounds varied by the fungal species; (3) competition among different species of fungi can affect both composition and concentration of FVOCs; (4) fungi can produce volatile compounds that can be attractive or inhibitive to bark beetles; (5) some symbiotic fungi are capable of transforming the primary MPB aggregation pheromone *trans*-verbenol into its anti-aggregation verbenone; (6) tree chemical defenses affect host suitability to bark beetles through influencing their fungal symbionts; and (7) different species of fungal symbionts respond differently to host defense metabolites.

Our research objectives are (1) to investigate the host terpene detoxification by MPBs’ fungal symbionts [38,44,45]; (2) to determine the benefits of maintaining multiple species of fungal symbionts to MPBs; (3) to test whether MPB elicits behavioral responses to FVOCs produced its fungal symbionts. Here, we inoculated the mature lodgepole pine trees in a forest stand with three fungal species (*G. clavigera, L. longiclavatum,* and *O. montium*) of MPB. To complement this field study, we inoculated the same fungal species on lodgepole pine logs in the laboratory. While live trees allow us to measure the time-specific interaction between trees and fungi, the log experiment allows measuring the host metabolites degradation process driven by the fungi. By collecting and analyzing the fungal-infected phloem samples, we identified and quantified the terpenes (monoterpenes, sesquiterpenes, and diterpenes) and the FVOC profile of each fungus. We then conducted an olfactometer assay to determine whether MPB is attracted to FVOCs associated with its symbiotic fungi through olfaction.

## 2. Methods

### 2.1. Field Phloem Sample Collection

We carried out a field experiment in lodgepole pine forests to characterize how different species of fungal symbionts of MPB alter the terpene chemistry of host phloem over time. We selected 10 healthy (asymptomatic) lodgepole pine trees (DBH = 25.05 ± 0.78 cm) at 22 km North-East of Hinton (Alberta; 53°30′50.7″ N 117°17′31.2″ W). On each tree, we open four holes 20 mm in size in four cardinal directions equidistant from each other at breast height (1.40 cm) along the tree stem. We placed 1 2 cm-sized plug of fungal mycelium (one of three fungal species) on each hole and 1 agar plug without fungal mycelium as control. The fungal plugs were taken from the edges of 10-day-old fungal cultures on potato dextrose agar media. Then, the wounds were covered with saran wraps. Phloem samples (from the fungal-infected and immediate upper part of the initial inoculation point, at different locations along the tree stems, i.e., 5–6 cm above the earlier sample) were collected after every 2 weeks for a total of 6 weeks, stored in dry ice in the field, brought to the laboratory, and stored at −40 °C until analysis. The tissues were processed and extracted based on the method described earlier [46]. The following fungi were used in this experiment; *G. clavigera* (EL004), *O. montium* (EL 031), and *L. longiclavatum* (EL002). Fungal cultures were obtained from different sources: *G. clavigera* was originally isolated from MPB in Fox Creek (Alberta) and provided by AV Rice (Northern Forestry Centre, Canadian Forest Service, Edmonton, Alberta), *L. longiclavatum* (NOF 3100) was provided by the Northern Forestry Centre Culture Collection, and *O. montium* (UAMH 4838) was provided by the University of Alberta Microfungus Collection and Herbarium (Edmonton).

### 2.2. Laboratory Experiment

The preliminary results from the above field study showed the highest induced terpene production in the phloem occurred at week 2; hence we further conducted a complementary laboratory experiment using logs of lodgepole pine trees. This study enabled us to better understand the host metabolite degradation process by fungi as well as to collect FVOCs. A total of 10 logs (21 × 30 cm: diameter × height) were selected. A 10 mm-sized plug of three fungi (as mentioned above) and 1 control (agar without fungal mycelium) were randomly inoculated on four cardinal directions of each log. Phloem samples were collected on day zero, during fungal inoculations, and 14 days post-inoculation and stored at − 40°C until analysis. After 14 days, fungal growth margins were traced, photographed, and used to quantify the culture area using ImageJ software version Java 1.8.0-172 (National Institutes of Health, Bethesda, MD, USA) [47].

Headspace volatiles from fungal-infected phloem samples were collected according to the method described in Cale et al. (2016) [13]. Briefly, infected tissues excised from logs were placed into a volatile collection chamber consisting of a 473 mL glass jar with Teflon tape on its threading and fitted with a metal cap. The jar was attached with a vacuum/pressure pump (Cole-Parmer Canada Inc., Montreal, QC, Canada). Constant airflow through chamber lines was set to 450 mL min^−1^ using a flowmeter. A Teflon tube filled with activated carbon (450 mg; 6–14 mesh, Fisher Sci., Hampton, NH, USA) fixed in place with glass wool was used to collect headspace volatiles from the jar for 6 h, after which time the carbon-filled tubes were removed from the collection apparatus Volatiles were extracted by adding the activated carbon to a microtube containing 1 mL of dichloromethane with tridecane as the internal standard (0.002%). This mixture was vortexed for 30 s, sonicated for 10 min, and centrifuged (at 18,213× *g*) for 30 min before the extract was collected and transferred to a 2 mL glass gas chromatography (GC) vial. This procedure was repeated a second time [14].

Phloem samples were processed as above and stored at −40 °C until further analysis [46].

### 2.3. Chemical Analysis

All extracts were analyzed using a GC fitted with a DB-5MS UI column (30 m × 0.25 mm ID × 0.25 μm film, product: 122-5532UI; Agilent Tech., Santa Clara, CA, USA) and coupled to a mass spectrometer (GC-MS; GC: 7890A, MS: 5062C, Agilent Tech.). Helium was used as a carrier gas flowing at 1 mL min^−1^ with a temperature program beginning at 45–50 °C (held for 2 min), followed by an increase of 3 °C min^−1^ to 70 °C, then 5 °C min^−1^ to 130 °C, after that 12 °C min^−1^ to 170 °C, and finally the column temperature was brought to 300 °C (held 2 min) at a rate of 30 °C min^−1^. A 1 μL sample injection volume was used; the injector temperature was 250 °C, and samples were run in splitless mode. The Sim and Scan acquisition mode was conducted simultaneously; while Sim mode allows us to acquire low traces of VOC and terpene compounds, Scan mode is performed for identification purposes. The NIST 2017 Mass Spectral library version 2.3 was used for the verification of all compounds. All compounds were quantified based on the following standards availability: *Monoterpenes*: limonene (Chem Purity: >99%, racemic mixture), β-pinene (CP: >99%, RM), β-myrcene (CP: 90%), α-pinene (CP: 98%, RM), β-phellandrene (CP: 96%, RM), α-phellandrene (CP: 95%), p-cymene (CP: >99%), terpinolene (CP: 90%), 3-carene (CP: 98.5%, RM), camphene (CP: 90%, RM), α-terpinene (CP: 85%), γ-terpinene (CP: 97%), ocimene (CP: 90%), *Oxygenated monoterpenes*: (-)-borneol (>99%), camphor (CP: 95%), α-terpineol (CP: 90%, RM), linalool (CP: 97%), *cis*-grandisol (CP: >95%), verbenone (CP: >99%), *Phenylpropenes*: 4-allylanisole (CP: 98.5%), Sesquiterpenes: (+) aromadendrene (CP: 97%), caryophyllene oxide (CP: 95%), β-caryophyllene (CP: 80%), *Aliphatics/others*: iso-butanol (CP: >99%), phenethyl alcohol (CP: >99%), 2-methyl-1-butanol (CP: >99%), phenethyl acetate (CP: >98%), 3-methyl-1-butanol (CP: >98%), iso-amyl acetate (CP: >97%), acetoin (CP: >96%),. All standards were obtained from Sigma-Aldrich (Oakville, ON, Canada), except β-phellandrene from TRC Canada (Toronto, ON, Canada). For the quantitation of some sesquiterpene compounds, due to their unavailability in the market, we used some of the above-mentioned standards to quantify based on hydrocarbon groups along with unique ion masses.

### 2.4. Two-Choice Olfactometer Test

We prepared a plant-based media as described earlier [16,38]: 7% lodgepole pine phloem powder and 4% bactoagar were mixed in 100 mL of distilled water. The powder supplemented fungal growth on the agar and made the diet palatable for adult MPBs. All three symbiotic fungi were grown on the media for 5 days. We developed a novel olfactometer setup that contained a 55 mm round Petri dish connected with 2 × 10 cm polyvinyl chloride tubes from opposite sides. The two tubes were further distally connected with two 15 mL falcon tubes (attached through the lid). The petri dish and the tubes were masked with vinyl electrical tape to make the experimental environment dark. As MPBs are positively phototactic insects, the whole setup was placed under a light source that was visible to beetles through the falcon tubes. A 6 mm fungal plug was placed inside either of the 2 falcon tubes. A media plug without fungus was inserted inside the other falcon tube that served as a control. A single adult female beetle was placed inside the Petri dish, and after 20 min, the beetle choice was recorded. Thus, a total of 20 beetles were tested for each fungal treatment. A beetle that did not respond to either treatment after 20 min was discarded from the experiment and replaced with another beetle. We used beetles that emerged from our mountain pine beetle colony in our bioassays; these beetles are reared on lodgepole pine logs.

### 2.5. Statistical Analysis

Data from fungal growth were transformed to Ln (Y) of original values to assure normality (Kolmogorov–Smirnov test). Then, Welch’s ANOVA test was done in GraphPad Prism version 9.0.0 for Windows (GraphPad Software, San Diego, CA, USA, www.graphpad.com, accessed on 15 July 2022). Dunnett’s T3 multiple comparison test was done to compare the means between different treatments (*p* < 0.05).

The final data matrices of chemical concentration from the field and laboratory studies were imported into the metabolomics data analysis platform Metaboanalyst 5.0 for data exploration, visualization, and multivariate statistical analysis (http://www.metaboanalyst.ca, accessed on 1 September 2022) [48]. Missing inputs were replaced by values calculated as half of the minimum positive values in the original data. The data were log2 transformed and auto-scaled to obtain a normal distribution.

The field study data were used for repeated measure analysis in live trees and were processed using the “Time-Series/One-factor” option. Principle component analysis (PCA) was applied to inspect the variation with averages of ten replicates from each treatment and each week. PERMANOVA test was performed to ascertain significant differences among different weeks, different treatment samples, and interaction between week and treatment, followed by Tukey’s multiple comparison test. Two-way repeated measure ANOVA, ANOVA-Simultaneous Component Analysis (ASCA) and multivariate empirical Bayes (MEBA) time-series analysis were performed to determine potential biomarkers that significantly regulated the host tree-fungal interaction.

To understand the dimensionality of the overall terpene chemical profile from the laboratory inoculations, we subjected the whole compound concentration dataset to Non-Metric Multidimensional Scaling (NMDS) indirect gradient analysis. PERMANOVA test was performed to ascertain the significant impact of treatments on terpene chemistry. Then for each fungal treatment, we performed fold change analysis coupled with 1-way ANOVA to estimate the individual compounds that showed at least 2-fold upregulation or downregulation compared to the control treatment. After that, multiple comparisons between different treatments for the specific compound were done by Fisher’s protected least significant difference (LSD). Correlations between fungal treatments and VOCs were calculated with the parametric test Pearson’s correlation.

Data from the 2-choice olfactometer assay were subjected to a 2-sample *t*-test. Significant differences were determined at *p* < 0.01. All the data were tested for normality assumption and homogeneity of variance before performing the statistical tests.

## 3. Results

### 3.1. Metabolic Profiles of Live P. contorta Trees Inoculated with Symbiotic Fungi

**Figure 1:**
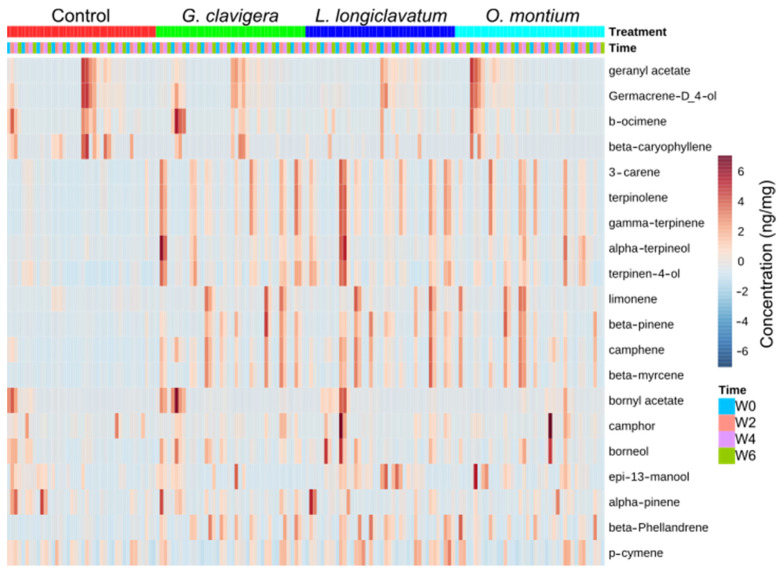
Combination of the heat map and hierarchical cluster analysis of the secondary metabolites in *Pinus contorta* phloem (treatment and control groups) during the period of weeks 0to 6 (*n* = 10). The color gradient panel on the right represents the highest to lowest concentrations from the darkest red to the darkest blue, respectively. Legends for time factor denoted by W0 = week 0, W2 = week 2, W4 = week 4 and W6 = week 6. Here, the distance was measured by the Euclidean method and clusters were prepared by the Ward clustering algorithm method.

We quantified 20 metabolites: monoterpenes (β-phellandrene, β-ocimene, 3-carene, terpinolene, limonene, β-pinene, camphene, β-myrcene, bornyl acetate, α-pinene, p-cymene, and geranyl acetate), sesquiterpenes (germacrene-d-4-ol, β-caryophyllene), diterpene (epi-13-manool), and oxygenated monoterpenes (α-terpineol, γ-terpinene, terpinen-4-ol, borneol, camphor). The metabolic profile of trees over 6 weeks was visualized by a heat map using the actual concentration of metabolites. The heat map provided interesting findings, including (1) inoculations with all fungal symbionts led to the induction of all of the host monoterpenes, relative to the control treatment; (2) the highest metabolite induction occurred at week 2 and thereafter, the concentrations decreased; (3) the concentrations of oxygenated monoterpenes increased by several folds following fungal inoculations compared to the control at weeks 2 and 4 (Figure 1; Tables S2–S5).

**Figure 2:**
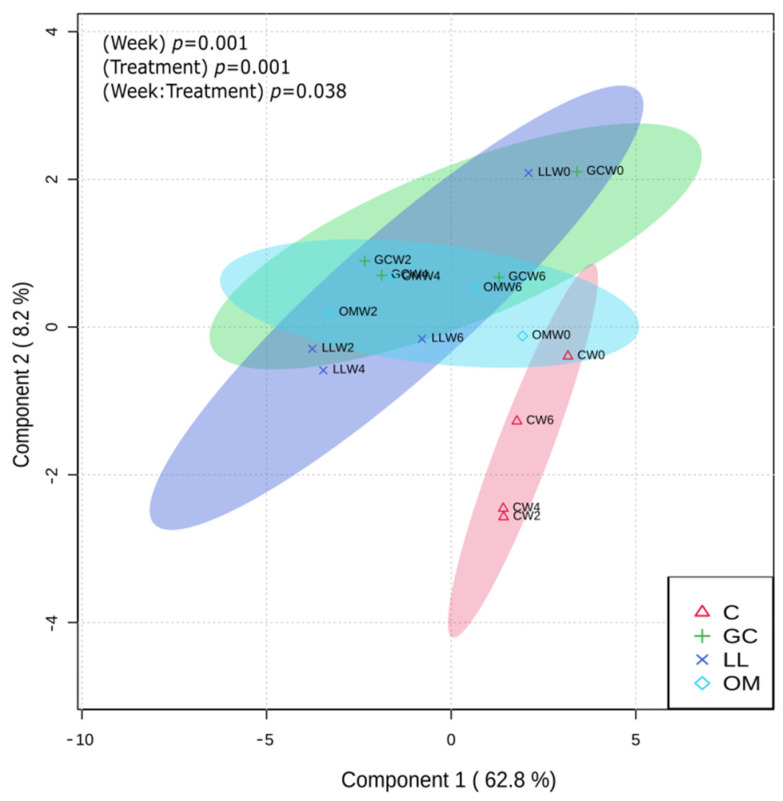
Principal component analysis of secondary metabolites of *Pinus contorta* inoculated with the three fungal symbionts of *Dendroctonus ponderosae*. C = control, GC = *Grosmannia clavigera*, LL = *Leptographium longiclavatum*, OM = *Ophiostoma montium*. The clusters of different treatments were denoted with different colors and 95% confidence interval eclipses. Significant differences among treatments were determined by PERMANOVA.

We performed PCA combined with PERMANOVA to investigate the effects of different explanatory variables on the changes in metabolite concentrations over time. The PCA 1 and 2 explained a total of 71% variance in the metabolite concentrations (Figure 2). While concentrations in control and fungal treatments at week 0 clustered closely, treatment clusters separated away from each other by weeks 2–6. Furthermore, the PERMANOVA test revealed significant differences in the metabolite concentrations over time (R^2^ = 0.33, F_3_ = 28.41, *p* = 0.001). Treatment and treatment x time interaction were also significant (F_3_ = 4.66, R^2^ = 0.05, *p* = 0.001 and F_9_ = 1.48, R^2^ = 0.05; *p* = 0.04 respectively; Figure 2).

We also conducted two-way repeated measure ANOVA (within subjects) to analyze which factors (time, treatment, and their interaction) caused differences among metabolites. Out of 20 metabolites, the concentrations of 5, 17, and 8 metabolites were affected by treatments, time, and their interaction, respectively (Table 1).

**Figure 3:**
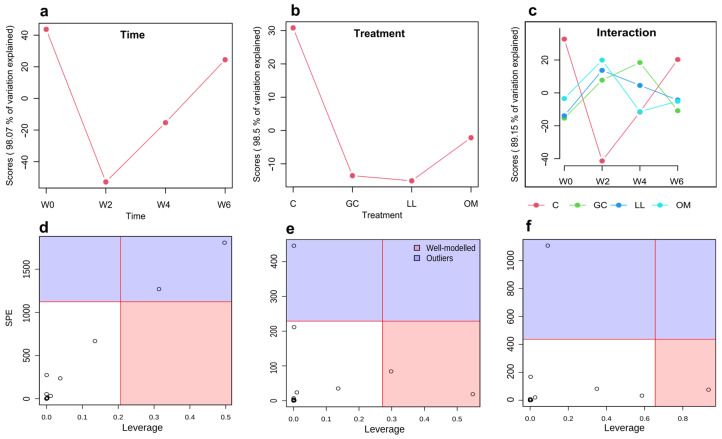
ANOVA-simultaneous component analysis (ASCA) of the induced secondary metabolites of *Pinus contorta* phloem following fungal inoculations of trees from weeks 0 to 6. (**a**–**c**) Major pattern related to time, treatments, and interaction between them; (**d**–**f**) important variables (metabolites) selected by ASCA related to time, treatments and their interaction respectively calculated by leverage/SPE analysis.

**Figure 4:**
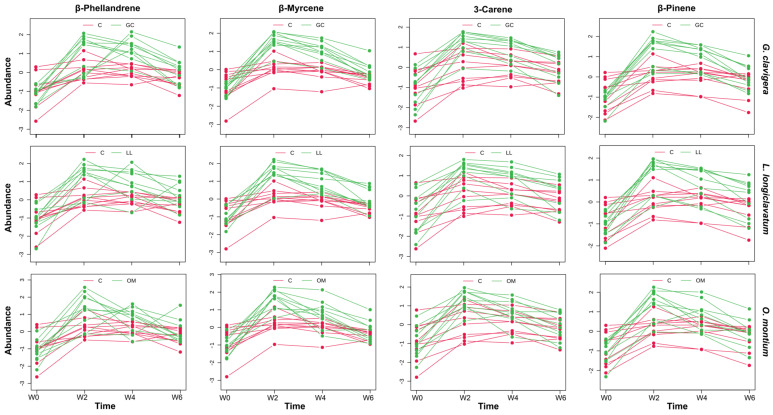
Multivariate empirical Bayes time-series analysis of *Pinus contorta* metabolites following inoculations by the fungal symbionts of *Dendroctonus ponderosae* selected based on two-way ANOVA, well-modeled by SPE and Hotelling’s T^2^ value > 10. Here, C = control; GC = *Grosmannia clavigera*; LL = *Leptographium longiclavatum*; OM = *Ophiostoma montium*.

Furthermore, we performed ASCA to ascertain the trends associated with different treatments, time, and their interactions [49]. The score plot for the time factor with component 1 (98.07% of variation explained) of the corresponding model showed a decrease in scores from week 0 to week 2, then increased at weeks 4 and 6 (Figure 3a). The score plot for the treatment factor showed that treatment types differed in their PC1 scores; the control treatment score was higher than those of fungal treatments, with *L. longiclavatum* having the lowest score (Figure 3b). The interaction effect score plot exhibited clear opposite trends at week 2 between treatments and control (Figure 3c). To correlate metabolic features with explanatory variables, we further constructed Leverage/squared prediction error (SPE) plots. Leverage assesses the importance of metabolites to the model, and SPE tests the model’s fitness for a particular metabolite. Well-modeled metabolites were selected based on high-leverage SPEs that contribute significantly to the model. The dots in the red area of Figure 3e correspond to β-myrcene, β-pinene while the dot in Figure 3f shows β-phellandrene. We then further conducted multivariate empirical Bayes (MEBA) time-series analysis to specify metabolic biomarkers that significantly (Hotelling T^2^ value > 10) shifted in comparison to the control (Table 1). Analyzing both models, four metabolites were selected out of 20, which were considered potential biomarkers (β-phellandrene, β-myrcene, β-pinene, and 3-carene; Table 1, Figure 4).

### 3.2. Kinetic Metabolic Pattern of Potential Biomarkers Following Fungal Infection

Following two-way ANOVA and ASCA, the four metabolites that were affected by either time or treatments or their interactions were further analyzed by MEBA to see the kinetic pattern change over time. Week 2 was the critical time point when the abundance of all four metabolites increased sharply (Figure 4). By weeks 4 and 6, the abundance of metabolites decreased gradually. In response to all three fungal inoculations, the abundance of β-phellandrene was increased up to four-fold at week 2 compared to the control (Hotelling’s T value > 20; F = 30.10, *p* < 0.05; Table 1, Figure 4). The abundance of β-myrcene in all fungal treatments significantly increased up to five-fold by week 2 compared to the control (time: F = 80.86, *p* < 0.001; treatment: F = 6.33, *p* < 0.05). Similarly, 3-carene was also significantly upregulated up to two-fold by week 2 compared to the control in all three fungal treatments (time: F = 57.41, *p* <0.001). The abundance of β-pinene was affected by both time and treatment (time: F = 43.31, *p* < 0.001; treatment: F = 5.31, *p* < 0.05; Figure 4, Table 1).

### 3.3. Effect of Fungal Inoculations on Chemotypic Traits of P. contorta Logs

**Figure 5:**
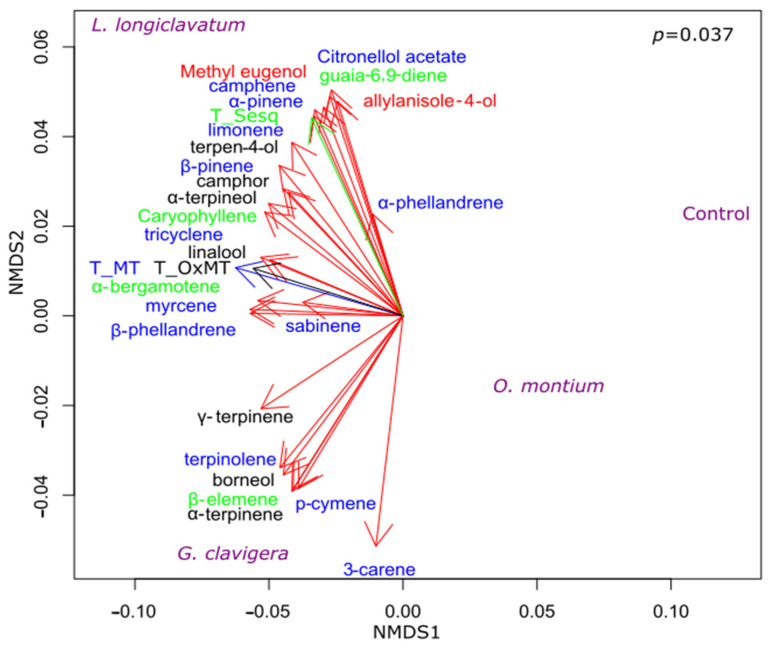
Effects of the inoculations by the fungal symbionts of *Dendroctonus ponderosae* on the terpene concentration of *Pinus contorta* phloem (logs). Individual terpene concentrations (ng mg^−1^ DW) were used in the analysis. Data were analyzed using NMDS gradient analysis. Treatments, monoterpenes, oxygenated monoterpenes, sesquiterpenes, and phenylpropanes were represented in violet, blue, black, green, and red, respectively (all red vectors). Significant differences between treatments were determined by PERMANOVA at *p* < 0.05. T_MT = Total Monoterpenes (blue vector), T_OxMT = Total Oxygenated Monoterpenes (black vector), T_sesq = Total Sesquiterpenes (green vector).

We performed NMDS on the metabolites collected from infected and non-infected phloem tissues of logs. The analysis was combined with the PERMANOVA test to further investigate the significance of treatment effects. We found that fungal inoculations significantly altered the metabolite concentrations (F = 4.66, *p* < 0.05; Figure 5). The total monoterpene and the total oxygenated monoterpene concentrations were correlated with both *G. clavigera* and *L. longiclavatum*, whereas the total sesquiterpene concentration was correlated with *L. longiclavatum*. Phenylpropenes such as methyl eugenol and allylanisole-4-ol were correlated with *L. longiclavatum* (Figure 5).

**Figure 6:**
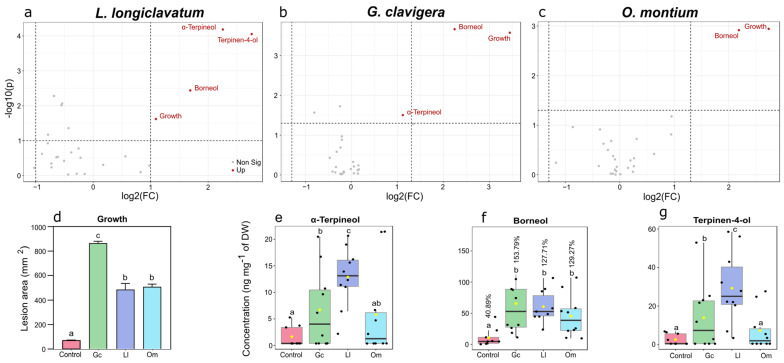
Upregulation of oxygenated monoterpenes in *Pinus contorta* phloem following inoculations by the fungal symbionts of *Dendroctonus ponderosae*. (**a**–**c**) Volcano plots show the fold change analysis of oxygenated monoterpenes combined with *t*-test (**a**) *Leptographium longiclavatum*, (**b**) *Grosmannia clavigera*, (**c**) *Ophiostoma montium* (Fold change analysis in *X*-axis, T-test in *Y*-axis; *p* < 0.05), (**d**) comparison of symbiotic fungal growth on the phloem, (**e**–**g**) upregulation of oxygenated monoterpenes such as (**e**) α-terpineol, (**f**) borneol, and (**g**) terpinen-4-ol by fungal infection (One-way ANOVA; followed Fisher’s LSD; *p* < 0.05), (**f**) proportional increment of borneol to bornyl acetate in the phloem tissues inoculated with symbiotic fungi compared to control after 14 days (One-way ANOVA followed by Fisher’s LSD test at *p* < 0.05). Significant differences (**d**–**g**) between different treatments are denoted by small letters.

Symbiotic fungi also differed in their virulence (F_3, 14.24_ = 135.50). Overall, *G. clavigera* induced the largest lesion (total fungal infected area on phloem) area compared to *L. longiclavatum* (*p* < 0.001), *O. montium* (*p* < 0.001), and control (*p* < 0.001) treatments. Both *L. longiclavatum* and *O. montium* had similar lesion areas (*p* > 0.99) while larger than the control (*p* < 0.001; Figure 6a–d). In pre-fungal inoculation samples, we detected a total of 23 compounds: α-pinene, camphene, β-myrcene, 3-carene, limonene, α-terpinene, p-cymene, γ-terpinene, terpinolene, linalool, β-phellandrene, α-phellandrene, β-pinene, α-terpineol, bornyl acetate, aromadendrene, allylanisole-4-ol, germacrene-d-4-ol, δ-cadinol, γ-cadinene, α-muurolene, guaia-6,9-diene, δ-cadinene. In 14 days post fungal inoculated samples, we detected additional 24 compounds that were not detected constitutively: tricyclene, borneol, camphor, terpen-4-ol, methyl eugenol, caryophyllene, α-bergamotene, β-elemene, citronellol acetate, acetoin, grandisol, isobutanol, phenethyl alcohol, verbenone, 3-methyl-1-butanol, 2-methyl-1-butanol, 2-methyl-2-butanol, 2,4-dimethyl-1-heptene, 4-methyl-octane, 4-methylheptane, 1-butanol, 3-methyl-2-butanone, 2-ethyl-1-butanol, 3,4-dimethoxyphenol (Table S1).

### 3.4. Symbiotic Fungi Influence the Proportion of Oxygenated Monoterpenes

To investigate whether the fungal inoculations can alter the concentration of oxygenated monoterpenes, we conducted a fold change analysis combined with a parametric t-test. Interestingly, all three fungi significantly upregulated borneol at least two-fold compared to the control (Figure 6a–c). *Leptographium longiclavatum* significantly upregulated α-terpineol up to four-fold (FC = 4.78, *p* < 0.001), and *G. clavigera* was upregulated two-fold (FC = 2.16, *p* < 0.05) relative to the control. Both *G. clavigera* and *L. longiclavatum* significantly increased the concentration of terpinen-4-ol compared to the control and *O. montium* (FC = 2.53 and 6.77, respectively; *p* < 0.05).

Logs inoculated with all three fungi caused a stronger proportional increment of borneol to bornyl acetate compared with the control, corresponding to an over three-fold increase (Figure 6f). Moreover, in the log experiment, we detected bornyl acetate at day 0 only before the fungal inoculations but not borneol and camphor, which were only detected on day 14 post-inoculation. In addition, there was a positive correlation between fungi-induced lesions and borneol (R^2^ = 0.5253, Spearman’s correlation, *p* < 0.001). Altogether, the oxygenated monoterpene concentration increased several folds post-fungal treatments (Figure S1).

### 3.5. Mountain Pine Beetles Were Attracted to Their Symbiotic Fungi

**Figure 7:**
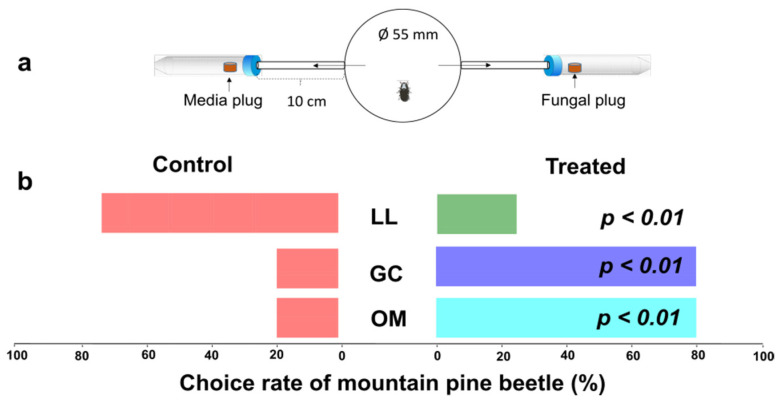
Behavioral responses of female *Dendroctonus ponderosae* to different fungal symbionts: (**a**) Experimental setup (described in methods). (**b**) The right bar graph shows beetle responses to fungal treatments, and the left bar graph shows beetle responses to controls. *p*-values show a significant difference at the 0.05 level by conducting a two-sample *t*-test. Here, LL = *Leptographium longiclavatum*, GC = *Grosmannia clavigera*, OM = *Ophiostoma montium*.

**Figure 8:**
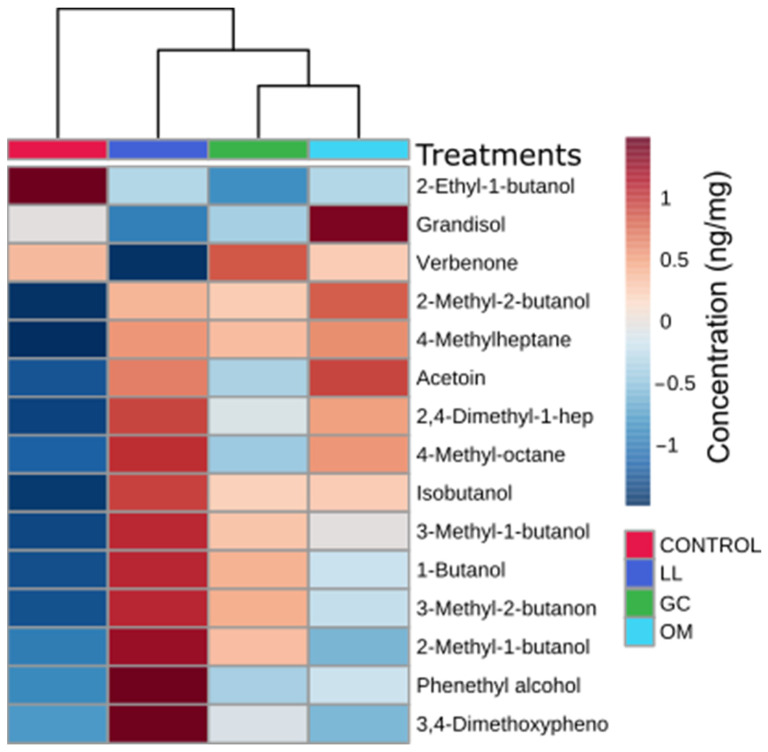
Heat map analysis, combined with hierarchical cluster analysis (HCA) of the fungal volatile organic compounds in *Pinus contorta* phloem (treatment and control groups) after 14 days (*n* = 10). The color gradient panel on the right represents metabolic abundance from the darkest red (high) to the darkest blue (low). Legends for treatment factor denoted by GC = *Grosmannia clavigera*, LL = *Leptographium longiclavatum*, OM = *Ophiostoma montium*. Here, the distance was measured by the Euclidean method and clusters were prepared by the Ward clustering algorithm method.

Two-choice olfactometer assay revealed significant results between control and fungal symbionts (Figure 7). Here, *G. clavigera* and *O. montium* responded similarly and attracted 80% of tested beetles as compared to the control (*p* < 0.01). In contrast, *L. longiclavatum* only attracted 25% of the tested beetle as compared to the control, while the remaining 75% showed attraction towards the control treatment (*p* < 0.01). Interestingly, concentrations of most of the FVOCs were comparatively higher in *L. longiclavatum* (Figure 8). The FVOC identified were as follows acetoin, grandisol, isobutanol, phenethyl alcohol, verbenone, 3-methyl-1-butanol, 2-methyl-1-butanol, 2-methyl-2-butanol, 2,4-dimethyl-1-heptene, 4-methyl-octane, 4-methylheptane, 1-butanol, 3-methyl-2-butanone, 2-ethyl-1-butanol, and 3,4-dimethoxyphenol (Figure 8, Table S1).

## 4. Discussion

We clearly show that MPB fungal symbionts can upregulate tree terpene defenses both in mature trees and logs and modify host monoterpenes to oxygenated derivatives in logs. Time seems to be a crucial factor in tree-induced responses, as the highest induction occurred two weeks after inoculations over the six-week duration of the experiment. Furthermore, the three fungal symbionts differed in their virulence as evidenced by differences in lesion lengths, the conversion efficiency of monoterpenes to oxygenated monoterpenes, and attraction to MPB *via* FVOCs. Together, these results demonstrate that MPB fungal symbionts play crucial roles during host colonization by bark beetles, including assisting beetles in the alteration of host tree defenses and likely increasing beetle attraction *via* the production of oxygenated monoterpenes and FVOCs [1,45,50]. Furthermore, both field and laboratory experiments provide complementary information that cannot be achieved by either alone.

### 4.1. Several Monoterpene Biomarkers Are Associated with Tree Responses to Fungal Inoculations

We show that low-density fungal inoculations can upregulate defense metabolites of lodgepole pine trees as fast as two weeks following inoculations, in agreement with earlier investigations in this [51,52,53] and other [54,55,56] study systems. However, not all terpenes were similarly upregulated as the concentrations of some of the monoterpenes and diterpenes were increased, while concentrations of all sesquiterpenes identified remained similar over the period of 6 weeks. Among monoterpenes, concentrations of β-phellandrene, β-myrcene, β-pinene, and 3-carene were several folds greater in the fungal inoculated trees, relative to the control, parallel to the results of other studies [29,57]. Some of these monoterpenes are reported to be highly toxic to MPB [58], supporting their importance in tree resistance. Among diterpenes, we found upregulation of 13-epi-manool which can suppress the reproduction and growth of fungal pathogens [59]. This is the first report of this labdane diterpenoid in response to the fungal inoculations in lodgepole pine. These results suggest that β-phellandrene, β-myrcene, β-pinene, 3-carene, and 13-epi-manool can be potential biomarkers and important components of the host chemical defenses against the fungal infection.

### 4.2. Conversion of Monoterpenes to Oxygenated Derivatives Appears to Be a Common Strategy to Reduce the Toxicity among Bark Beetle

Conifer monoterpenes are toxic to several species of bark beetles, including MPB [58,60,61,62,63,64,65], and their conversion to oxygenated derivatives may lessen their toxicity. For example, α-pinene and myrcene were reported to be more toxic than their oxygenated derivatives, bornyl acetate and linalool, respectively, to bark beetles [58,60,66]. Therefore, the conversion of monoterpenes to their less toxic oxygenated derivatives by fungal symbionts could reduce the extent of monoterpene toxicity to MPB. Earlier studies reported a similar detoxification mechanism by fungal symbionts of several bark beetle species [10,44]. Auto-oxidation of monoterpenes can also occur as the resin encounter air; however, in this study, we found the concentrations of several oxygenated monoterpenes such as borneol and terpinene-4-ol were multiple folds higher in the fungal treated tissues compared to the control, suggesting the possible role of fungi in the detoxification process. In support of this, Wang et al. [11] reported that *G. clavigera* contains genes encoding cytochromes P450 and several other oxidative enzymes that can degrade and utilize monoterpenes, such as limonene. Interestingly, some of the oxygenated monoterpenes, such as borneol, were reported to elicit attraction in MPB [17].

### 4.3. Fungal-Produced Volatile Organic Compounds Serve as Attractant Cues for Beetles

Fungal symbionts of bark beetles are reported *de novo* synthesis of VOCs that act as attractants for several species of bark beetles [13,15,16]. Here, we demonstrated a close-range attraction of MPBs to VOCs of their fungal symbionts. Among them, phenethyl alcohol was reported as an attractant for MPB in field tests [17]. These results complement the vast literature on the attractiveness of FVOCs in other bark beetle species [39,67,68,69]. For instance, the fungal volatiles 2-phenylethyl acetate and 3-methyl-1-butyl acetate increased the attraction of *D. frontalis* to its pheromone blend [70]. Similarly, *I. typographus* utilizes the fungal volatile 2-methyl-3-buten-2-ol as aggregation pheromone [16,30]; other FVOCs released from the same fungal symbionts improved the attraction of *I. typographus* to its pheromone [18,38]. Furthermore, the walnut twig beetle, *Pityophthorus juglandis*, was attracted to the FVOCs produced by its primary bark fungi [71]. In the current study, we did not detect all FVOCs reported in our earlier studies [13,14,40], probably due to differences in the timing of volatile collection between studies. Nevertheless, our olfactometer experiment reveals that MPB can recognize their fungal symbionts by detecting their FVOCs. Interestingly, not all three fungal species tested were attractive to MPB, as 80% of adult MPB tested were attracted to *G. clavigera* and *O. montium*, but only 25% of beetles were attracted to *L. longiclavatum*. Such differences in attraction may be attributed to the greater abundance of most of the FVOCs associated with *L. longiclavatum* relative to those of *G. clavigera* and *O. montium*.

## 5. Conclusions

We demonstrate that fungal symbionts of MPB can upregulate host tree defense metabolites and convert monoterpenes to oxygenated derivatives. Through this mechanism, fungi can help beetles to exhaust and deplete terpene defenses, enabling beetles to overcome host resistance and making host substrates suitable for larval growth [51]. Maintaining multiple symbionts provides the beetles with a variety of benefits, including nutritional supplementation, protection and many other complementary benefits. We further propose that *de novo* synthesized FVOCs volatiles and oxygenated monoterpenes may improve the attraction of bark beetles to trees during host colonization. However, FVOCs and oxygenated monoterpenes can be attractive or repellent to beetles depending on their specific concentrations. Whether FVOCs elicit behavioral responses in bark beetles should be verified in the field experiment. Nevertheless, FVOCs and oxygenated monoterpenes can be potential components in integrated pest management strategies to control the bark beetle population [15,18,71]. The potential tree resistance biomarkers we have identified in our study can be used in tree breeding through genomic approaches to generate more resistant trees to beetle-fungal attack.

## Figures and Tables

**Table 1:** Metabolite profile of *Pinus contorta* phloem inoculated with the fungal symbionts of *Dendroctonus ponderosae*. Biomarkers are selected based on statistical significance in two-way ANOVA and well-modeled with Hotelling’s T2 value > 10.

	Treatment			Time			Interaction			Hotelling-T^2^		
Metabolites	F	p*	*p*^	F	p*	*p*^	F	p*	*p*^	G	Ll	Om
β-Myrcene	6.333	0.001	0.026	80.864	1.69 × 10^−27^	3.39 × 10^−26^	6.085	6.93 × 10^−7^	1.39 × 10^−5^	27.102	17.5	15.052
β-Pinene	5.311	0.004	0.026	43.314	1.86 × 10^−18^	5.31 × 10^−18^	4.387	6.72 × 10^−5^	0.001	23.415	21.095	13.926
Camphene	5.077	0.005	0.026	71.673	1.39 × 10^−25^	1.39 × 10^−24^	4.9301	1.52 × 10^−5^	0.000	8.676	5.779	5.2164
β-Phellandrene	5.043	0.005	0.026	30.098	3.20 × 10^−14^	6.41 × 10^−14^	2.994	0.003	0.010	61.257	25.006	44.444
γ-Terpinene	3.172	0.036	0.143	57.656	2.49 × 10^−22^	1.14 × 10^−21^	3.653	0.001	0.003	1.309	0.8404	0.616
3-Carene	2.831	0.052	0.173	57.416	2.86 × 10^−22^	1.14 × 10^−21^	3.348	0.001	0.005	23.153	18.273	13.56
Terpinolene	2.654	0.063	0.181	49.326	3.69 × 10^−20^	1.23 × 10^−19^	2.975	0.003	0.010	16.078	11.555	9.421
Terpinen-4-ol	2.083	0.120	0.299	34.55	9.82 × 10^−16^	2.46 × 10^−15^	2.196	0.028	0.055	0.039	0.025	0.019
Limonene	1.924	0.143	0.318	22.144	2.99 × 10^−11^	5.43 × 10^−11^	2.533	0.011	0.025	10.265	8.910	9.106
Borneol	1.769	0.171	0.341	34.235	1.25 × 10^−15^	2.77 × 10^−15^	2.675	0.008	0.019	0.014	0.014	0.009
p-Cymene	1.349	0.274	0.456	68.208	8.04 × 10^−25^	5.36 × 10^−24^	1.217	0.292	0.450	0.021	0.103	0.021
α-Pinene	1.206	0.321	0.494	15.264	2.39 × 10^−8^	3.67 × 10^−8^	1.842	0.069	0.125	11.495	8.122	4.828
α-Terpineol	1.036	0.388	0.537	18.548	8.90 × 10^−10^	1.48 × 10^−9^	1.040	0.413	0.551	0.180	0.078	0.063
Camphor	0.959	0.423	0.537	14.371	6.05 × 10^−8^	8.64 × 10^−8^	1.130	0.348	0.498	0.001	0.001	0.001
epi-13-Manool	0.944	0.430	0.537	3.202	0.026	0.031	1.359	0.221	0.368	3.295	4.982	1.629
Germacrene-D-4-ol	0.284	0.837	0.877	5.457	0.002	0.002	0.581	0.811	0.897	3.313	1.627	1.228
Bornyl acetate	0.227	0.877	0.877	4.914	0.003	0.004	0.528	0.852	0.897	1.369	1.187	0.709

p*: raw *p*-value; *p*^: adjusted *p*-value; Gc: *Grosmannia clavigera*, Ll: *Leptographium longiclavatum*, Om: *Ophiostoma montium*.

## Data Availability

The data presented in this study are available on request from the corresponding author. The data are not publicly available due to the corresponding author’s need for further research.

## Supplementary Materials

The following supporting information can be downloaded at: https://www.mdpi.com/article/10.3390/metabo13020239/s1. Figure S1: Pie chart representing the chemical composition of *Pinus contorta* phloem (cut logs) before and after 14 days of fungal inoculation. Table S1: Emission of volatile organic compounds identified from the headspace collection of fresh pine bark, fourteen days after inoculation with different fungi. Analyses were conducted using GC-MS. Table S2: Relative amounts (mean ± SE, n = 10) of volatiles from uninfected bark detected after various time periods (0, 14, 28 and 42 days) from the beginning of an experiment. Data from the control uninfected treatment are presented here. Table S3: Relative amounts (mean ± SE, n = 10) of volatiles from uninfected bark detected after various time periods (0, 14, 28 and 42 days) from the beginning of an experiment. Data from the control *G. clavigera* treatment are presented here. Table S4: Relative amounts (mean ± SE, n = 10) of volatiles from uninfected bark detected after various time periods (0, 14, 28 and 42 days) from the beginning of an experiment. Data from the control *L. longiclavatum* treatment are presented here. Table S5: Relative amounts (mean ± SE, n = 10) of volatiles from uninfected bark detected after various time periods (0, 14, 28 and 42 days) from the beginning of an experiment. Data from the control *O. montium* treatment are presented here.

## References

[1] Frago E., Dicke M., Godfray H.C.J. (2012). Insect Symbionts as Hidden Players in Insect-Plant Interactions. Trends Ecol. Evol..

[2] Blomquist G.J., Figueroa-Teran R., Aw M., Song M., Gorzalski A., Abbott N.L., Chang E., Tittiger C. (2010). Pheromone Production in Bark Beetles. Insect Biochem. Mol. Biol..

[3] VitÉ J.P., Pitman G.B. (1968). Bark Beetle Aggregation: Effects of Feeding on the Release of Pheromones in *Dendroctonus* and *Ips*. Nature.

[4] Zhao T., Axelsson K., Krokene P., Borg-Karlson A.K. (2015). Fungal Symbionts of the Spruce Bark Beetle Synthesize the Beetle Aggregation Pheromone 2-Methyl-3-Buten-2-Ol. J. Chem. Ecol..

[5] Safranyik L., Wilson B. (2007). The Mountain Pine Beetle: A Synthesis of Biology, Management, and Impacts on Lodgepole Pine.

[6] Six D.L. (2012). Ecological and Evolutionary Determinants of Bark Beetle—Fungus Symbioses. Insects.

[7] Six D.L. (2013). The Bark Beetle Holobiont: Why Microbes Matter. J. Chem. Ecol..

[8] Wadke N., Kandasamy D., Vogel H., Lah L., Wingfield B.D., Paetz C., Wright L.P., Gershenzon J., Hammerbacher A. (2016). Catechol Dioxygenases Catalyzing the First Step in Norway Spruce Phenolic Degradation Are Key Virulence Factors in the Bark Beetle-Vectored Fungus *Endoconidiophora polonica*. Plant Physiol..

[9] Zhao T., Kandasamy D., Krokene P., Chen J., Gershenzon J., Hammerbacher A. (2019). Fungal Associates of the Tree-Killing Bark Beetle, *Ips typographus*, Vary in Virulence, Ability to Degrade Conifer Phenolics and Influence Bark Beetle Tunneling Behavior. Fungal Ecol..

[10] Hammerbacher A., Schmidt A., Wadke N., Wright L.P., Schneider B., Bohlmann J., Brand W.A., Fenning T.M., Gershenzon J., Paetz C. (2013). A Common Fungal Associate of the Spruce Bark Beetle Metabolizes the Stilbene Defenses of Norway Spruce. Plant Physiol..

[11] Wang Y., Lim L., Madilao L., Lah L., Bohlmann J., Breuil C. (2014). Gene Discovery for Enzymes Involved in Limonene Modification or Utilization by the Mountain Pine Beetle-Associated Pathogen *Grosmannia clavigera*. Appl. Environ. Microbiol..

[12] Lehenberger M., Foh N., Göttlein A., Six D., Biedermann P.H.W. (2021). Nutrient-Poor Breeding Substrates of Ambrosia Beetles Are Enriched with Biologically Important Elements. Front. Microbiol..

[13] Cale J.A., Collignon R.M., Klutsch J.G., Kanekar S.S., Hussain A., Erbilgin N. (2016). Fungal Volatiles Can Act as Carbon Sources and Semiochemicals to Mediate Interspecific Interactions among Bark Beetle-Associated Fungal Symbionts. PLoS ONE.

[14] Cale J.A., Ding R., Wang F., Rajabzadeh R., Erbilgin N. (2019). Ophiostomatoid Fungi Can Emit the Bark Beetle Pheromone Verbenone and Other Semiochemicals in Media Amended with Various Pine Chemicals and Beetle-Released Compounds. Fungal Ecol..

[15] Kandasamy D., Gershenzon J., Hammerbacher A. (2016). Volatile Organic Compounds Emitted by Fungal Associates of Conifer Bark Beetles and Their Potential in Bark Beetle Control. J. Chem. Ecol..

[16] Kandasamy D., Gershenzon J., Andersson M.N., Hammerbacher A. (2019). Volatile Organic Compounds Influence the Interaction of the Eurasian Spruce Bark Beetle (*Ips typographus*) with Its Fungal Symbionts. ISME J..

[17] Pureswaran D.S., Gries R., Borden J.H., Pierce H.D. (2000). Dynamics of Pheromone Production and Communication in the Mountain Pine Beetle, *Dendroctonus ponderosae* Hopkins, and the Pine Engraver, *Ips pini* (Say) (Coleoptera: Scolytidae). Chemoecology.

[18] Jirošová A., Modlinger R., Hradecký J., Ramakrishnan R., Beránková K., Kandasamy D. (2022). Ophiostomatoid Fungi Synergize Attraction of the Eurasian Spruce Bark Beetle, *Ips typographus* to Its Aggregation Pheromone in Field Traps. Front. Microbiol..

[19] Wermelinger: B. (2004). Ecology and Management of the Spruce Bark Beetle *Ips typographus*—A Review of Recent Research. For. Ecol. Manag..

[20] Raffa K.F., Aukema B.H., Bentz B.J., Carroll A.L., Hicke J.A., Turner M.G., Romme W.H. (2008). Cross-Scale Drivers of Natural Disturbances Prone to Anthropogenic Amplification: The Dynamics of Bark Beetle Eruptions. Bioscience.

[21] Bentz B.J., Rgnire J., Fettig C.J., Hansen E.M., Hayes J.L., Hicke J.A., Kelsey R.G., Negron J.F., Seybold S.J. (2010). Climate Change and Bark Beetles of the Western United States and Canada: Direct and Indirect Effects. Bioscience.

[22] Bohlmann J., Gershenzon J., Aubourg S. (2000). Biochemical, Molecular, Genetic and Evolutionary Aspects of Defense-Related Terpenoid Metabolism in Conifers. Evolution of Metabolic Pathways.

[23] Franceschi V.R., Krokene P., Christiansen E., Krekling T. (2005). Anatomical and Chemical Defenses of Conifer Bark against Bark Beetles and Other Pests. New Phytol..

[24] Keeling C.I., Bohlmann J. (2006). Genes, Enzymes and Chemicals of Terpenoid Diversity in the Constitutive and Induced Defence of Conifers against Insects and Pathogens. New Phytol..

[25] Bohlmann J. (2008). Insect-Induced Terpenoid Defenses in Spruce. Induced Plant Resistance to Herbivory.

[26] Krokene P., Vega Fernando E., Hofstetter R.W. (2015). Conifer Defense and Resistance to Bark Beetles. Bark Beetles: Biology and Ecology of Native and Invasive Species.

[27] Raffa K.F., Berryman A.A. (1983). The Role of Host Plant Resistance in the Colonization Behavior and Ecology of Bark Beetles (Coleoptera: Scolytidae). Ecol. Monogr..

[28] Klepzig K.D., Smalley E.B., Raffa K.F. (1996). Combined Chemical Defenses against an Insect-Fungal Complex. J. Chem. Ecol..

[29] Ullah A., Klutsch J.G., Erbilgin N. (2021). Production of Complementary Defense Metabolites Reflects a Co-Evolutionary Arms Race between a Host Plant and a Mutualistic Bark Beetle-Fungal Complex. Plant Cell Environ..

[30] Raffa K.F., Andersson M.N., Schlyter F. (2016). Host Selection by Bark Beetles: Playing the Odds in a High-Stakes Game. Adv. Insect Phys..

[31] Erbilgin N., Mori S.R., Sun J.H., Stein J.D., Owen D.R., Merrill L.D., Bolaños R.C., Raffa K.F., Montiel T.M., Wood D.L. (2007). Response to Host Volatiles by Native and Introduced Populations of *Dendroctonus valens* (Coleoptera: Curculionidae, Scolytinae) in North America and China. J. Chem. Ecol..

[32] Klimetzek D., Francke W. (1980). Relationship between the Enantiomeric Composition of α-Pinene in Host Trees and the Production of Verbenols in *Ips* Species. Experientia.

[33] Lanne B.S., Ivarsson P., Johnsson P., Bergström G., Wassgren A.B. (1989). Biosynthesis of 2-Methyl-3-Buten-2-Ol, a Pheromone Component of *Ips typographus* (Coleoptera: Scolytidae). Insect Biochem..

[34] Bleiker K.P., Potter S.E., Lauzon C.R., Six D.L. (2009). Transport of Fungal Symbionts by Mountain Pine Beetles. Can. Entomol..

[35] Lee S., Kim J.J., Breuil C. (2006). Diversity of Fungi Associated with the Mountain Pine Beetle, *Dendroctonus ponderosae* and Infested Lodgepole Pines in British Columbia. Fungal Divers..

[36] Roe A.D., James P.M.A., Rice A.V., Cooke J.E.K., Sperling F.A.H. (2011). Spatial Community Structure of Mountain Pine Beetle Fungal Symbionts Across a Latitudinal Gradient. Microb. Ecol..

[37] Erbilgin N. (2019). Phytochemicals as Mediators for Host Range Expansion of a Native Invasive Forest Insect Herbivore. New Phytol..

[38] Kandasamy D., Zaman R., Nakamura Y., Zhao T., Hartmann H., Andersson M.N., Hammerbacher A., Gershenzon J. (2021). Bark Beetles Locate Fungal Symbionts by Detecting Volatile Fungal Metabolites of Host Tree Resin Monoterpenes. bioRxiv.

[39] Hulcr J., Mann R., Stelinski L.L. (2011). The Scent of a Partner: Ambrosia Beetles Are Attracted to Volatiles from Their Fungal Symbionts. J. Chem. Ecol..

[40] Wang F., Cale J.A., Hussain A., Erbilgin N. (2020). Exposure to Fungal Volatiles Can Influence Volatile Emissions from Other Ophiostomatoid Fungi. Front. Microbiol..

[41] Guevara-Rozo S., Hussain A., Cale J.A., Klutsch J.G., Rajabzadeh R., Erbilgin N. (2020). Nitrogen and Ergosterol Concentrations Varied in Live Jack Pine Phloem following Inoculations with Fungal Associates of Mountain Pine Beetle. Front. Microbiol..

[42] Liu Y., Anastacio G.R., Ishangulyyeva G., Rodriguez-Ramos J.C., Erbilgin N. (2021). Mutualistic Ophiostomatoid Fungi Equally Benefit from Both a Bark Beetle Pheromone and Host Tree Volatiles as Nutrient Sources. Microb. Ecol..

[43] Agbulu V., Zaman R., Ishangulyyeva G., Cahill J.F., Erbilgin N. (2021). Host Defense Metabolites Alter the Interactions between a Bark Beetle and Its Symbiotic Fungi. Microb. Ecol..

[44] Nones S., Sousa E., Holighaus G. (2022). Symbiotic Fungi of an Ambrosia Beetle Alter the Volatile Bouquet of Cork Oak Seedlings. Phytopathology.

[45] Westrick N.M., Smith D.L., Kabbage M. (2021). Disarming the Host: Detoxification of Plant Defense Compounds During Fungal Necrotrophy. Front. Plant Sci..

[46] Erbilgin N., Ma C., Whitehouse C., Shan B., Najar A., Evenden M. (2014). Chemical Similarity between Historical and Novel Host Plants Promotes Range and Host Expansion of the Mountain Pine Beetle in a Naïve Host Ecosystem. New Phytol..

[47] Schindelin J., Arganda-Carreras I., Frise E., Kaynig V., Longair M., Pietzsch T., Cardona A. (2012). Fiji: An Open-Source Platform for Biological-Image Analysis. Nature Methods.

[48] Xia J., Psychogios N., Young N., Wishart D.S. (2009). MetaboAnalyst: A Web Server for Metabolomic Data Analysis and Interpretation. Nucleic Acids Res..

[49] He Y., Wang Y., Hu C., Sun X., Li Y., Xu N. (2019). Dynamic Metabolic Profiles of the Marine Macroalga Ulva Prolifera during Fragmentation-Induced Proliferation. PLoS ONE.

[50] Lieutier F., Yart A., Salle A. (2009). Stimulation of Tree Defenses by Ophiostomatoid Fungi Can Explain Attack Success of Bark Beetles on Conifers. Ann. For. Sci.

[51] Kim J., Plattner A., Lim Y., Breuil C. (2008). Comparison of Two Methods to Assess the Virulence of the Mountain Pine Beetle Associate, *Grosmannia clavigera*, to *Pinus contorta*. Scand. J. For. Res..

[52] Plattner A., Kim J.-J., Diguistini S., Breuil C. (2008). Variation in Pathogenicity of a Mountain Pine Beetle-Associated Blue-Stain Fungus, *Grosmannia clavigera*, on Young Lodgepole Pine in British Columbia. Can. J. Plant Pathol..

[53] Rice A.V., Thormann M.N., Langor D.W. (2007). Mountain Pine Beetle Associated Blue-Stain Fungi Cause Lesions on Jack Pine, Lodgepole Pine, and Lodgepole x Jack Pine Hybrids in Alberta. Can. J. Bot..

[54] Zhao T., Krokene P., Hu J., Christiansen E., Björklund N., Långström B., Solheim H., Borg-Karlson A.K. (2011). Induced Terpene Accumulation in Norway Spruce Inhibits Bark Beetle Colonization in a Dose-Dependent Manner. PLoS ONE.

[55] Ben Jamaa M.L., Lieutier F., Yart A., Jerraya A., Khouja M.L. (2007). The Virulence of Phytopathogenic Fungi Associated with the Bark Beetles *Tomicus piniperda* and *Orthotomicus erosus* in Tunisia. For. Pathol..

[56] Brignolas F., Lieutier F., Sauvard D., Yart A., Drouet A., Claudot A.C. (1995). Changes in Soluble-phenol Content of Norway-spruce (*Picea abies*) Phloem in Response to Wounding and Inoculation with *Ophiostoma polonicum*. Eur. J. For. Pathol..

[57] Cale J.A., Klutsch J.G., Dykstra C.B., Peters B., Erbilgin N. (2019). Pathophysiological Responses of Pine Defensive Metabolites Largely Lack Differences between Pine Species but Vary with Eliciting Ophiostomatoid Fungal Species. Tree Physiol..

[58] Chiu C.C., Keeling C.I., Bohlmann J. (2017). Toxicity of Pine Monoterpenes to Mountain Pine Beetle. Sci. Rep..

[59] Cheng S.S., Chung M.J., Lin C.Y., Wang Y.N., Chang S.T. (2012). Phytochemicals from *Cunninghamia konishii* Hayata Act as Antifungal Agents. J. Agric. Food Chem..

[60] Everaerts C., Grégoire J.-C., Merlin J. (1988). The Toxicity of Norway Spruce Monoterpenes to Two Bark Beetle Species and Their Associates. Mechanisms of Woody Plant Defenses Against Insects.

[61] Reid M.L., Sekhon J.K., LaFramboise L.M. (2017). Toxicity of Monoterpene Structure, Diversity and Concentration to Mountain Pine Beetles, *Dendroctonus ponderosae*: Beetle Traits Matter More. J. Chem. Ecol..

[62] Coats J.R., Karr L.L., Drewes C.D. (1991). Toxicity and Neurotoxic Effects of Monoterpenoids. Am. Chem. Soc. Symp. Ser..

[63] Werner R.A. (1995). Toxicity of Repellency of 4-Allylanisole and Monoterpenes from White Spruce and Tamarack to the Spruce Beetle and Eastern Larch Beetle (Coleoptera: Scolytidae). Environ. Entomol..

[64] Scalerandi E., Flores G.A., Palacio M., Defagó M.T., Carpinella M.C., Valladares G., Bertoni A., Palacios S.M. (2018). Understanding Synergistic Toxicity of Terpenes as Insecticides: Contribution of Metabolic Detoxification in *Musca domestica*. Front. Plant Sci..

[65] Regnault-Roger C., Hamraoui A. (1995). Fumigant Toxic Activity and Reproductive Inhibition Induced by Monoterpenes on *Acanthoscelides obtectus* (Say) (Coleoptera), a Bruchid of Kidney Bean (*Phaseolus vulgaris* L.). J. Stored Prod. Res..

[66] Seth Davis T., Stewardship R. (2020). Toxicity of Two Engelmann Spruce (*Pinaceae*) Monoterpene Chemotypes from the Southern Rocky Mountains to North American Spruce Beetle (Coleoptera: Scolytidae). Can. Entomol..

[67] Saerens S.M.G., Delvaux F.R., Verstrepen K.J., Thevelein J.M. (2010). Production and Biological Function of Volatile Esters in *Saccharomyces Cerevisiae*. Microb. Biotechnol..

[68] Christiaens J.F., Franco L.M., Cools T.L., de Meester L., Michiels J., Wenseleers T., Hassan B.A., Yaksi E., Verstrepen K.J. (2014). The Fungal Aroma Gene ATF1 Promotes Dispersal of Yeast Cells through Insect Vectors. Cell Rep..

[69] Luna E., Cranshaw W., Tisserat N. (2014). Attraction of Walnut Twig Beetle *Pityophthorus juglandis* (Coleoptera: Curculionidae) to the Fungus *Geosmithia morbida*. Plant Heal. Prog..

[70] Brand J.M., Schultz J., Barras S.J., Edson L.J., Payne T.L., Hedden R.L. (1977). Bark-Beetle Pheromones Enhancement of *Dendroctonus frontalis* (Coleoptera: Scolytidae) Aggregation Pheromone by Yeast Metabolites in Laboratory Bioassays. Chem. Ecol..

[71] Davis T.S., Crippen T.L., Hofstetter R.W., Tomberlin J.K. (2013). Microbial Volatile Emissions as Insect Semiochemicals. J. Chem. Ecol..

