# Supplementary material for: Bark Beetles Utilize Ophiostomatoid Fungi to Circumvent Host Tree Defenses
Source: Metabolites. 2023 Feb 6;13(2):239. doi: 10.3390/metabo13020239 (PMC9968207; doi:10.3390/metabo13020239)
Supplement: Supplementary file 1 [file metabolites-13-00239-s001.zip › metabolites-2159816-supplementary/Supp figures.pdf]

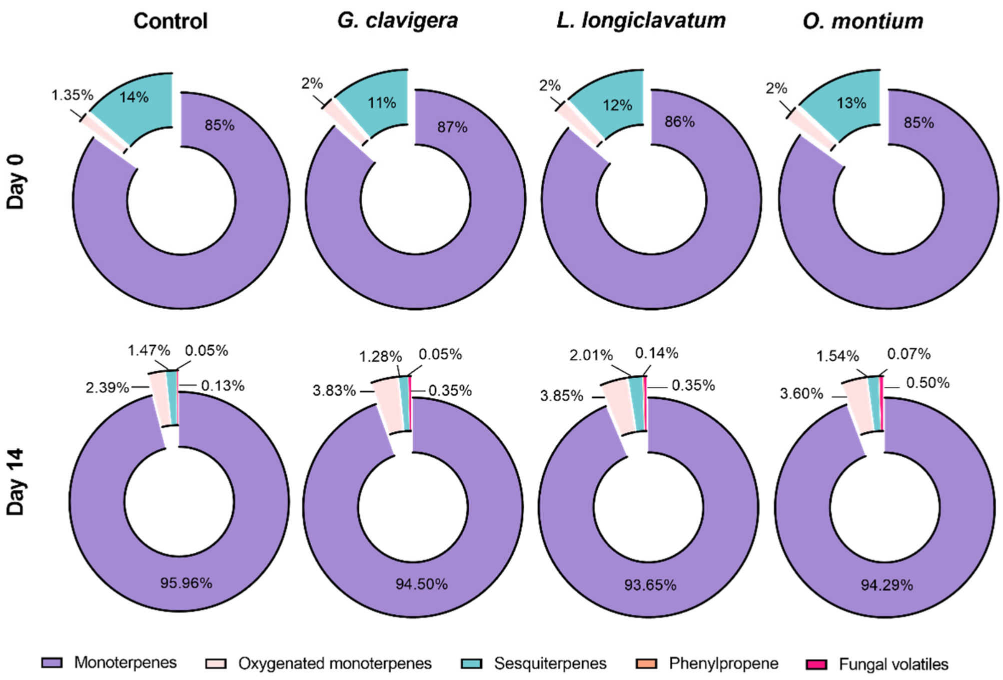

Figure S1: Pie chart representing the chemical composition of *Pinus contorta* phloem (cut logs) before and after 14 days of fungal inoculation.
