# Supplementary material for: Bark Beetles Utilize Ophiostomatoid Fungi to Circumvent Host Tree Defenses
Source: Metabolites. 2023 Feb 6;13(2):239. doi: 10.3390/metabo13020239 (PMC9968207; doi:10.3390/metabo13020239)
Supplement: Supplementary file 1 [file metabolites-13-00239-s001.zip › metabolites-2159816-supplementary/Supplementary tables.pdf]

Table S1: Emission of volatile organic compounds identified from the headspace collection of fresh pine bark, fourteen days after inoculation with different fungi. Analyses were conducted using GC-MS.

	RT[#]	Concentrations of compounds (ng per mg dry weight of bark)							
		Uninfected		*G. clavigera*		*L. longiclavatum*		*O. montium*	
Monoterpenes		Day 0	Day 14	Day 0	Day 14	Day 0	Day 14	Day 0	Day 14
Tricyclene	8.8	ND	0.51 ± 0.51	ND	2.14 ± 1.15	ND	3.69 ± 2.54	ND	2.11 ± 1.12
α-Pinene	9.24	225.1 ± 79.3	284.40 ± 60.98	173.90 ± 40.85	370 ± 55.29	224.20 ±68.63	784.20 ± 247.10	149.80 ± 31.81	393.70 ± 94.95
Camphene	9.91	36.59 ± 13.61	40.09 ± 8.93	25.71 ± 4.55	49.62 ± 7.90	28.95 ±6.67	95.28 ± 27.86	21.13 ± 3.44	47.92 ± 9.36
Sabinene	10.96	ND	7.76 ± 2.43	ND	8.38 ± 2.92	ND	9.25 ± 2.67	ND	9.24 ± 3.08
ß-Pinene	11.09	131.60 ± 38.24	221.50 ± 31.54	113.70 ± 20.44	297.70 ± 27.74	145.70 ±34.82	440.10 ± 110.60	97.39 ± 15.04	325.40 ± 58.52
Myrcene	11.64	48.52 ± 7.34	110.90 ± 26.76	60.46 ± 10.65	163.40 ± 49.49	69.36 ±12.52	182.90 ± 35.19	47.77 ± 6.84	138.20 ± 38.24
3-Carene	12.36	108.4 ± 32.34	610.80 ± 217.10	190.40 ± 65.65	888.40 ± 338.20	206.70 ±71.57	590.20 ± 214.60	154.60 ± 44.12	684.40 ± 266.80
α-Terpinene	12.71	8.53 ± 1.05	15.21 ± 3.94	21.85 ± 3.89	23.19 ± 7.14	17.28 ±3.69	21.63 ± 4.69	11.91 ± 2.18	19.67 ± 6.72
α-Phellandrene	12.29	37.46 ± 4.72	99.29 ± 38.85	78.18 ± 14.76	106.10 ± 38.65	69.87 ±14.56	103.60 ± 20.63	46.05 ± 7.86	64.78 ± 21.35
Cymene	13.05	2.40 ± 1.47	14.36 ± 3.74	1.748 ± 0.58	21.19 ± 5.60	1.96 ±0.47	19.52 ± 3.54	0.97 ± 0.41	17.45 ± 4.63
Limonene	13.19	131 ± 36.29	230.70 ± 33.71	121 ± 12.20	320.40 ± 58.26	146.20 ±24.67	510.20 ± 115.20	102.50 ± 12.58	291.10 ± 48.00
ß-Phellandrene	13.25	657.80 ± 72.69	1621 ± 416.00	834.90 ± 155.80	2292 ± 695.00	972.50 ±177.60	2530 ± 496.50	686.30 ± 104.40	1933 ± 546.40
γ-Terpinene	14.32	4.21 ± 0.60	11.64 ± 3.28	6.82 ± 1.51	18.52 ± 6.15	6.93 ±1.63	19.60 ± 3.43	5.09 ± 0.98	16.14 ± 5.31
Terpinolene	15.30	13.71 ± 3.19	45.00 ± 15.31	23.11 ± 6.98	71.61 ± 27.59	24.80 ±7.86	69.44 ± 16.83	18.21 ± 4.44	60.66 ± 23.94
Phenylproprenes									
Allylanisole-4-ol	19.12	11.86 ± 1.73	17.44 ± 6.54	8.31 ± 1.09	17.28 ± 4.12	10.39 ±1.96	28.50 ± 5.36	8.96 ± 0.72	20.02 ± 5.33
Methyl eugenol	24.46	ND	1.59 ± 0.78	ND	2.19 ± 0.85	ND	7.67 ± 4.53	ND	2.89 ± 1.50
Oxygenated monoterpenes									
Camphor	11.92	ND	1.87 ± 1.05	ND	3.73 ± 1.42	ND	7.67 ± 2.43	ND	4.96 ± 1.32
Linalool	15.88	1.83 ± 0.15	2.37 ± 0.47	2.49 ± 0.67	4.13 ± 1.45	3.05 ±0.86	6.21 ± 2.37	2.48 ± 0.36	4.72 ± 1.99
Borneol	18.30	ND	10.55 ± 4.79	ND	65.39 ± 16.58	ND	60.55 ± 7.93	ND	45.30 ± 10.66
Terpinen-4-ol	18.6	ND	2.38 ± 1.03	ND	13.78 ± 5.47	ND	29.29 ± 5.89	ND	7.07 ± 3.35
α-Terpineol	19.03	0	1.38 ± 0.71	0.83 ± 0.57	6.56 ± 2.40	1.14 ±0.76	12.88 ± 1.76	0.75 ± 0.52	5.64 ± 2.73
Verbenone	19.49	ND	0.44 ± 0.13	ND	0.51 ± 0.18	ND	0.17 ± 0.09	ND	0.42 ± 0.24
Bornyl acetate	21.71	23.23 ± 8.46	ND	42.52 ± 16.08	ND	47.41 ±18.36	ND	35.05 ± 10.53	ND

Citronellyl acetate	23.40	ND	0.01 ± 0.01	ND	0.01 ± 0.01	ND	0.05 ± 0.02	ND	0.01 ± 0.00
Sesquiterpenes									
ß-Elemene	24.31	ND	5.30 ± 2.57	ND	8.10 ± 3.09	ND	7.93 ± 3.88	ND	7.77 ± 3.38
ß-Caryophyllene	24.85	ND	3.44 ± 1.60	ND	6.07 ± 2.45	ND	8.85 ± 2.68	ND	5.31 ± 2.13
α-Bergamotene	25.08	ND	8.60 ± 3.29	ND	12.47 ± 3.97	ND	15.32 ± 4.26	ND	12.44 ± 4.20
Aromadendrene	25.96	6.61 ± 0.72	ND	14.72 ± 3.36	ND	18.32 ±4.43	ND	12.68 ± 2.01	ND
Germacrene-4-ol	26.91	49.11 ± 10.03	ND	26.87 ± 9.04	ND	23.96 ±6.54	ND	35.46 ± 7.89	ND
γ-Cadinene	26.24	35.87 ± 6.60	ND	41.10 ± 13.80	ND	50.26 ±10.61	ND	34.53 ± 4.64	ND
δ-Cadinol	27.24	22.13 ± 3.78	ND	18.89 ± 4.84	ND	31.81 ±6.45	ND	19.32 ± 3.08	ND
Guaia-6,9-diene	25.21	16.70 ± 6.67	32.72 ± 9.17	10.76 ± 3.69	35.30 ± 8.95	14.56 ±5.58	82.02 ± 25.35	10.46 ± 3.09	38.84 ± 9.32
δ-Cadinene	26.30	69.49 ± 12.90	ND	76.71 ± 7.97	ND	93.30 ±20.00	ND	71.48 ± 12.05	ND
α-Muurolene	26.05	19.78 ± 3.67	ND	26.01 ± 4.52	ND	28.48 ±6.18	ND	19.86 ± 2.77	ND
Aliphatics/ others									
Isobutanol	2.32	ND	0.00 ± 0	ND	1.92 ± 0.11	ND	2.65 ± 0.17	ND	1.93 ± 0.11
3-Methyl-2-butanone	2.55	ND	0.50 ± 0.08	ND	0.57 ± 0.02	ND	0.60 ± 0.03	ND	0.55 ± 0.02
1-Butanol	2.593	ND	0.53 ± 0.13	ND	0.96 ± 0.08	ND	1.12 ± 0.38	ND	0.79 ± 0.34
2-Methyl-2-butanol	2.9	ND	11.90 ± 1.57	ND	14.93 ± 0.73	ND	15.19 ± 0.62	ND	15.94 ± 1.07
Acetoin	3.13	ND	2.91 ± 1.25	ND	3.75 ± 0.84	ND	4.71 ± 0.39	ND	4.97 ± 0.86
3-Methyl-1-butanol	3.54	ND	0.50 ± 0.12	ND	1.07 ± 0.32	ND	1.35 ± 0.32	ND	0.96 ± 0.18
2-Methyl-butanol	3.65	ND	0.27 ± 0.07	ND	0.71 ± 0.13	ND	1.01 ± 0.19	ND	0.41 ± 0.09
4-Methylheptane	4.087	ND	3.44 ± 0.42	ND	4.23 ± 0.28	ND	4.31 ± 0.25	ND	4.33 ± 0.23
2,4-Dimethyl-1-heptene	5.911	ND	6.02 ± 0.57	ND	6.46 ± 0.43	ND	6.83 ± 0.49	ND	6.67 ± 0.29
4-Methyloctane	6.68	ND	0.36 ± 0.03	ND	0.37 ± 0.03	ND	0.39 ± 0.03	ND	0.39 ± 0.02
2-Ethyl-1-butanol	9.802	ND	0.73 ± 0.06	ND	0.67 ± 0.01	ND	0.68 ± 0.02	ND	0.68 ± 0.02
Phenethyl alcohol	16.37	ND	0.10 ± 0.02	ND	0.23 ± 0.07	ND	0.68 ± 0.19	ND	0.27 ± 0.05
Grandisol	19.6	ND	0.28 ± 0.09	ND	0.19 ± 0.08	ND	0.04 ± 0.03	ND	0.63 ± 0.22
3,4-Dimethoxyphenol	24.841	ND	0.08 ± 0.07	ND	0.19 ± 0.11	ND	0.45 ± 0.18	ND	0.11 ± 0.07

#RT: Retention time according to GC-MS analysis; ND: Not detected.

Table S2: Relative amounts (mean ± SE, n=10) of volatiles from uninfected bark detected after various time periods (0, 14, 28 and 42 days) from the beginning of an experiment. Data from the control uninfected treatment are presented here.

Uninfected (Control)		Concentrations of compounds (ng per mg dry weight of bark)			
Monoterpenes	RT#	Week 0	Week 2	Week 4	Week 6
α-Pinene	9.240	1.722 ± 0.798	25.258 ± 8.169	19.375 ± 7.447	4.332 ± 1.724
Camphene	9.910	0.169 ± 0.086	0.401 ± 0.135	0.285 ± 0.083	0.133 ± 0.018
ß-Pinene	11.090	1.532 ± 0.524	5.604 ± 1.938	4.497 ± 0.920	2.535 ± 0.457
ß-Myrcene	11.640	0.339 ± 0.081	0.976 ± 0.207	0.719 ± 0.089	0.401 ± 0.039
3-Carene	12.360	3.690 ± 1.892	14.862 ± 5.296	9.408 ± 2.880	5.273 ± 1.551
p-Cymene	13.050	0.041 ± 0.017	0.196 ± 0.016	0.141 ± 0.022	0.074 ± 0.014
Limonene	13.190	1.397 ± 0.813	2.224 ± 0.750	2.348 ± 0.909	1.222 ± 0.407
ß-Phellandrene	13.250	11.539 ± 2.971	28.471 ± 5.539	24.734 ± 2.477	17.572 ± 2.137
γ-Terpinene	14.320	0.049 ± 0.015	0.155 ± 0.045	0.124 ± 0.028	0.071 ± 0.016
Terpinolene	15.300	0.321 ± 0.108	1.126 ± 0.382	0.831 ± 0.219	0.390 ± 0.094
Oxygenated monoterpenes					
Geranyl acetate	11.62	1.149 ± 0.774	1.087 ± 0.732	0.957 ± 0.524	0.734 ± 0.440
Camphor	11.92	0.005 ± 0.003	0.016 ± 0.006	0.007 ± 0.002	0.001 ± 0.000
ß-ocimene	13.9	0.004 ± 0.003	0.006 ± 0.004	0.005 ± 0.002	0.005 ± 0.002
Borneol	18.3	0.021 ± 0.009	0.034 ± 0.012	0.034 ± 0.012	0.011 ± 0.003
Terpinen-4-ol	18.6	0.015 ± 0.006	0.018 ± 0.007	0.028 ± 0.009	0.017 ± 0.004
α-Terpineol	19.03	0.015 ± 0.004	0.049 ± 0.012	0.040 ± 0.006	0.019 ±0.005
Bornyl acetate	21.71	0.458 ± 0.376	0.599 ± 0.491	0.455 ± 0.316	0.115 ± 0.060
Sesquiterpenes					
ß-Caryophyllene	24.85	0.011 ± 0.005	0.015 ± 0.007	0.011 ± 0.006	0.008 ± 0.004
Germacrene-4-ol	26.91	3.705 ± 2.387	4.388 ± 2.570	2.979 ± 1.686	2.039 ± 1.260
Diterpenes					
Epi-13-manool	23.4	4.478 ± 1.533	3.398 ± 2.071	3.598 ± 1.292	2.255 ± 0.852

Table S3: Relative amounts (mean ± SE, n=10) of volatiles from uninfected bark detected after various time periods (0, 14, 28 and 42 days) from the beginning of an experiment. Data from the control *G. clavigera* treatment are presented here.

G. clavigera		Concentrations of compounds (ng per mg dry weight of bark)			
Monoterpenes	RT#	Week 0	Week 2	Week 4	Week 6
α-Pinene	9.240	0.823 ± 0.368	27.440 ± 7.648	11.421 ± 3.579	6.477 ± 1.800
Camphene	9.910	0.119 ± 0.064	2.380 ± 0.294	1.164 ± 0.196	0.320 ± 0.095
ß-Pinene	11.090	0.665 ± 0.096	49.835 ± 12.396	23.910 ± 5.040	5.301 ± 1.742
ß-Myrcene	11.640	0.216 ± 0.025	6.220 ± 0.881	3.157 ± 0.539	0.838 ± 0.220
3-Carene	12.360	2.244 ± 0.812	75.335 ± 14.993	40.151 ± 8.915	10.229 ± 2.528
p-Cymene	13.050	0.025 ± 0.009	0.163 ± 0.028	0.150 ± 0.026	0.067 ± 0.014
Limonene	13.190	0.758 ± 0.304	14.796 ± 5.271	8.222 ± 2.998	2.353 ± 1.136
ß-Phellandrene	13.250	8.074 ± 0.989	86.073 ± 18.769	91.077 ± 15.564	30.374 ± 7.018
γ-Terpinene	14.320	0.033 ± 0.008	0.769 ± 0.141	0.480 ± 0.095	0.127 ± 0.025
Terpinolene	15.300	0.234 ± 0.073	5.985 ± 1.246	3.683 ± 0.833	0.786 ± 0.176
Oxygenated monoterpenes					
Geranyl acetate	11.62	0.825 ± 0.417	0.924 ± 0.464	0.660 ± 0.270	0.761 ± 0.405
Camphor	11.92	0.001 ± 0.001	0.015 ± 0.004	0.010 ± 0.004	0.004 ± 0.001
ß-ocimene	13.9	0.003 ± 0.002	0.005 ± 0.005	0.005 ± 0.004	0.007 ± 0.003
Borneol	18.3	0.012 ± 0.004	0.080 ± 0.016	0.049 ± 0.009	0.012 ± 0.005
Terpinen-4-ol	18.6	0.008 ±0.002	0.086 ± 0.023	0.096 ± 0.017	0.032 ± 0.006
α-Terpineol	19.03	0.014 ± 0.003	0.210 ± 0.081	0.183 ± 0.053	0.033 ± 0.005
Bornyl acetate	21.71	0.431 ± 0.365	1.238 ± 0.818	0.751 ± 0.415	0.342 ± 0.258
Sesquiterpenes					
ß-Caryophyllene	24.85	0.005 ± 0.003	0.002 ± 0.002	0.008 ± 0.006	0.004 ± 0.004
Germacrene-4-ol	26.91	2.751 ± 1.307	3.545 ± 1.782	2.123 ± 0.852	2.411 ± 1.194
Diterpenes					
Epi-13-manool	23.4	2.160 ± 1.358	5.922 ± 3.384	1.952 ± 1.168	3.890 ± 1.593

Table S4: Relative amounts (mean ± SE, n=10) of volatiles from uninfected bark detected after various time periods (0, 14, 28 and 42 days) from the beginning of an experiment. Data from the control *L. longiclavatum* treatment are presented here.

L. longiclavatum		Concentrations of compounds (ng per mg dry weight of bark)			
Monoterpenes	RT[#]	Week 0	Week 2	Week 4	Week 6
α-Pinene	9.240	0.979 ± 0.362	19.792 ± 9.739	14.361 ± 6.510	11.136 ± 5.035
Camphene	9.910	0.106 ± 0.028	2.172 ± 0.406	1.185 ± 0.266	0.350 ± 0.073
ß-Pinene	11.090	0.831 ± 0.195	47.351 ± 10.580	24.518 ± 5.711	8.724 ± 2.667
ß-Myrcene	11.640	0.260 ± 0.054	6.003 ± 1.021	3.188 ± 0.678	0.990 ± 0.226
3-Carene	12.360	4.255 ± 1.953	74.198 ± 15.568	43.261 ± 12.191	14.523 ± 4.648
p-Cymene	13.050	0.024 ± 0.006	0.225 ± 0.025	0.206 ± 0.035	0.125 ± 0.034
Limonene	13.190	0.713 ± 0.284	13.665 ± 4.926	7.464 ± 2.933	2.350 ± 0.676
ß-Phellandrene	13.250	10.270 ± 1.978	97.382 ± 19.394	72.063 ± 16.036	36.702 ± 7.663
γ-Terpinene	14.320	0.054 ± 0.019	0.739 ± 0.151	0.524 ± 0.147	0.182 ± 0.053
Terpinolene	15.300	0.403 ± 0.166	5.850 ± 1.295	4.071 ± 1.239	1.146 ± 0.369
Oxygenated monoterpenes					
Geranyl acetate	11.62	0.755 ± 0.430	0.667 ± 0.333	0.464 ± 0.204	0.372 ± 0.177
Camphor	11.92	0.001 ± 0.000	0.023 ± 0.011	0.011 ± 0.004	0.002 ± 0.001
ß-ocimene	13.9	0.002 ± 0.002	0.001 ± 0.001	0.001 ± 0.001	0.005 ± 0.001
Borneol	18.3	0.013 ± 0.003	0.098 ± 0.028	0.046 ± 0.010	0.013 ± 0.003
Terpinen-4-ol	18.6	0.010 ± 0.005	0.093 ± 0.020	0.088 ± 0.023	0.040 ± 0.010
α-Terpineol	19.03	0.016 ± 0.005	0.182 ± 0.058	0.175 ± 0.072	0.056 ± 0.018
Bornyl acetate	21.71	0.288 ± 0.174	0.721 ± 0.476	0.779 ± 0.501	0.325 ± 0.178
Sesquiterpenes					
ß-Caryophyllene	24.85	0.007 ± 0.004	0.000 ± 0.000	0.006 ± 0.004	0.001 ± 0.001
Germacrene-4-ol	26.91	2.574 ± 1.382	3.005 ± 1.668	1.405 ± 0.491	1.372 ± 0.572
Diterpenes					
Epi-13-manool	23.4	7.779 ± 3.364	10.623 ± 3.266	3.709 ± 1.642	4.952 ± 1.761

Table S5: Relative amounts (mean ± SE, n=10) of volatiles from uninfected bark detected after various time periods (0, 14, 28 and 42 days) from the beginning of an experiment. Data from the control *O. montium* treatment are presented here.

O. montium		Concentrations of compounds (ng per mg dry weight of bark)			
Monoterpenes	RT#	Week 0	Week 2	Week 4	Week 6
α-Pinene	9.240	1.060 ± 0.442	12.922 ± 2.750	7.571 ± 1.868	10.412 ± 6.355
Camphene	9.910	0.085 ± 0.027	1.926 ± 0.330	0.875 ± 0.298	0.249 ± 0.093
ß-Pinene	11.090	0.773 ± 0.161	43.294 ± 11.255	18.546 ± 7.326	4.355 ± 1.701
ß-Myrcene	11.640	0.230 ± 0.047	5.400 ± 0.909	2.371 ± 0.745	0.664 ± 0.190
3-Carene	12.360	2.522 ± 1.127	63.853 ± 14.157	25.926 ± 7.179	7.758 ± 2.243
p-Cymene	13.050	0.023 ± 0.007	0.167 ± 0.033	0.150 ± 0.033	0.054 ± 0.011
Limonene	13.190	0.757 ± 0.313	13.448 ± 4.894	7.066 ± 3.418	1.923 ± 0.984
ß-Phellandrene	13.250	8.648 ± 1.732	97.719 ± 22.419	48.542 ± 8.344	25.756 ± 7.314
γ-Terpinene	14.320	0.036 ± 0.011	0.639 ± 0.125	0.311 ± 0.082	0.101 ± 0.027
Terpinolene	15.300	0.263 ± 0.106	4.968 ± 1.102	2.360 ± 0.691	0.609 ± 0.169
Oxygenated monoterpenes					
Geranyl acetate	11.62	1.036 ± 0.782	0.982 ± 0.648	0.938 ± 0.540	0.627 ± 0.298
Camphor	11.92	0.002 ± 0.001	0.023 ± 0.012	0.007 ± 0.003	0.002 ± 0.001
ß-ocimene	13.9	0.004 ± 0.004	0.002 ± 0.002	0.001 ± 0.001	0.005 ± 0.001
Borneol	18.3	0.011 ± 0.003	0.071 ± 0.022	0.032 ± 0.012	0.009 ± 0.003
Terpinen-4-ol	18.6	0.008 ± 0.002	0.071 ± 0.019	0.063 ± 0.013	0.028 ± 0.007
α-Terpineol	19.03	0.020 ± 0.008	0.167 ± 0.050	0.119 ± 0.043	0.044 ± 0.016
Bornyl acetate	21.71	0.167 ± 0.108	0.455 ± 0.306	0.241 ± 0.104	0.144 ± 0.073
Sesquiterpenes					
ß-Caryophyllene	24.85	0.006 ± 0.005	0.000 ±0.000	0.004 ± 0.004	0.004 ± 0.003
Germacrene-4-ol	26.91	3.271 ± 2.105	2.973 ± 1.877	2.511 ± 1.436	1.790 ± 0.857
Diterpenes					
Epi-13-manool	23.4	6.207 ± 2.451	7.840 ± 4.578	3.608 ± 1.369	3.055 ± 1.847
